# Primary Non-Hodgkin Lymphoma of the Urinary Bladder in the Paediatric Age Group: A Report of a Rare Case

**DOI:** 10.7759/cureus.91033

**Published:** 2025-08-26

**Authors:** Rakesh Kumar, Kashif Rizwi, Pradosh Kumar Sarangi, Tarun Kumar, Bibin Sunny

**Affiliations:** 1 Urology, All India Institute of Medical Sciences, Patna, Patna, IND; 2 Radiodiagnosis, All India Institute of Medical Sciences, Patna, Patna, IND; 3 Radiodiagnosis, All India Institute of Medical Sciences, Deoghar, Deoghar, IND; 4 Pathology/Laboratory Medicine, All India Institute of Medical Sciences, Patna, Patna, IND

**Keywords:** bladder tumour, burkitt lymphoma, contrast-enhanced computed tomography (cect), cop chemotherapy, copdam chemotherapy, french-american-british (fab) classification, ki-67 marker, non-hodgkin lymphoma

## Abstract

Primary non-Hodgkin lymphoma (NHL) of the urinary bladder in the paediatric age group is an unusual neoplasm. Patients mostly present with haematuria, lower urinary tract symptoms and abdominal pain. The clinical, radiological and histological findings are non-specific. It is difficult to differentiate it from more common congeners in this age bracket, such as rhabdomyosarcoma, on the basis of imaging. The diagnosis is made on the basis of histopathological characteristics and is confirmed by immunohistochemical (IHC) analysis. Herein, we report a case of a six-year-old male child who presented with lower urinary tract symptoms and abdominal pain for a duration of two months. He was further evaluated using cross-sectional imaging, and histopathological examination with immunohistochemistry confirmed the diagnosis of NHL (Burkitt lymphoma). He was treated with one cycle of COP (cyclophosphamide, oncovin and prednisone), followed by one cycle of COPDAM (cyclophosphamide, oncovin, prednisone, doxorubicin (Adriamycin) and methotrexate). Follow-up contrast-enhanced computed tomography (CECT) revealed a 60% reduction in tumour size. This case highlights the importance of early diagnosis based on histopathology and IHC, and demonstrates that timely initiation of appropriate chemotherapy can lead to favourable outcomes in NHL.

## Introduction

The genitourinary system is rarely involved by lymphoma, and about 6%-8% of patients will manifest urological symptoms by genitourinary extension of aggressive lymphoma [1]. Non-Hodgkin lymphoma (NHL) is one of the most aggressive tumours [2]. This tumour proliferates early and is associated with tumour lysis syndrome [2]. The prognosis of lymphoma depends largely on the histological subtype and stage of disease. With advancements in diagnostic modalities and chemotherapy, the overall outcome of this tumour has improved significantly. Herein, we describe a rare case of primary NHL of the urinary bladder in a six-year-old boy.

## Case presentation

**Figure 1:**
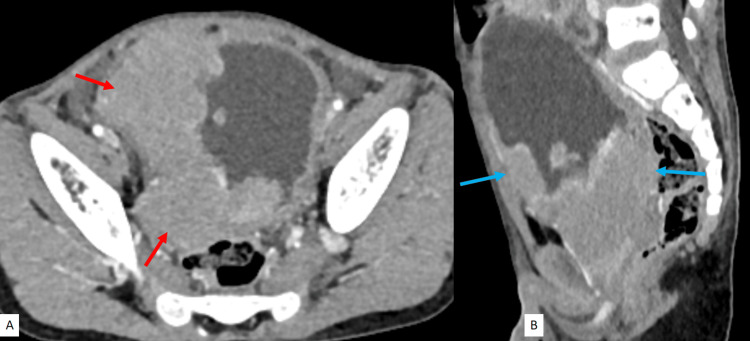
CECT scan of the abdomen: (A) Axial section showing a homogeneously enhancing mass arising from the right lateral wall and trigone of the urinary bladder (red arrows). (B) Sagittal section demonstrating anterior and posterior perivesical extensions of the mass (blue arrows). CECT: contrast-enhanced computed tomography

A six-year-old boy came to the urology outpatient department with complaints of frequency, urgency and lower abdominal pain for a two-month duration. He had no significant past medical or family history. On clinical examination, approximately 6×4 cm, hard, smooth, non-mobile mass was palpable in the suprapubic region with ill-defined borders. Routine urine analysis and urine culture were performed, which showed no evidence of macroscopic or microscopic haematuria, and the culture was sterile. Initial ultrasonography of the abdomen showed an irregular hypoechoic mass arising from the urinary bladder, involving bilateral vesicoureteral junction, leading to bilateral moderate hydroureteronephrosis. Further evaluation with contrast-enhanced computed tomography (CECT) of the abdomen was done, which revealed a heterogeneously enhancing lobulated lesion measuring of size 9×5×7 cm involving the right lateral wall and trigone of the urinary bladder with bilateral vesicoureteral junction involvement (Figure 1A). It also showed extravesical extensions, anteriorly to the recti and posteriorly to the mesorectal fascia and rectum (Figure 1B).

**Figure 2:**
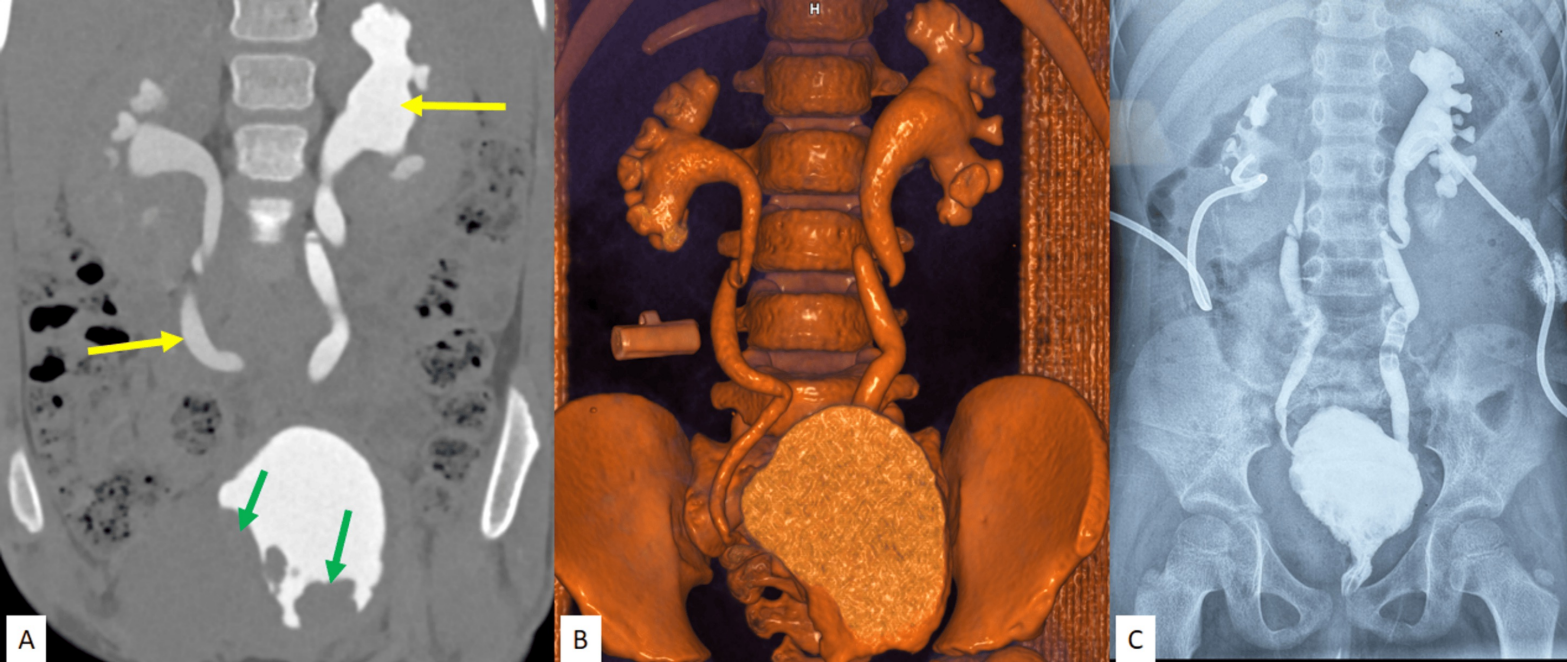
(A) Excretory phase of CECT scan (coronal section) showing a filling defect in the bladder lumen (green arrows) and contrast retention in bilateral dilated pelvicalyceal systems and ureters (yellow arrows). (B) 3D VRT image demonstrating bilateral moderate hydroureteronephrosis. (C) Bilateral percutaneous nephrostogram images. CECT: contrast-enhanced computed tomography, VRT: volume-rendered technique

**Figure 3:**
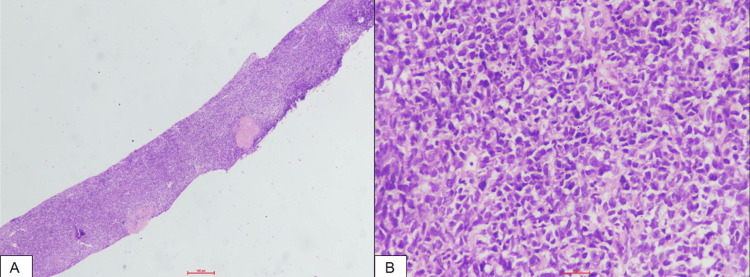
(A and B): Urinary bladder wall showing diffuse infiltration by monomorphic round-to-oval tumour cells with hyperchromatic nuclei and scant cytoplasm (haematoxylin and eosin stain; A: 4×, B: 40×).

**Figure 4:**
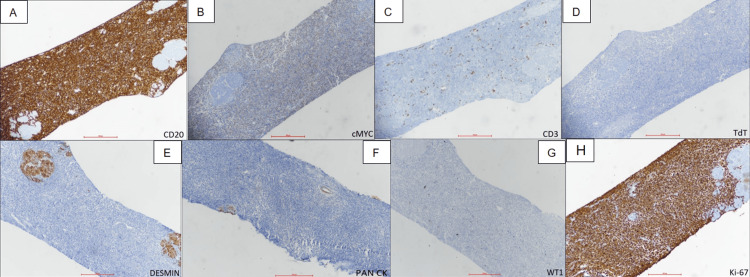
(A-H): Immunohistochemistry showing positive expression of CD20 (A) and C-MYC (B). Tumour cells are negative for CD3 (C), TdT (D), desmin (E), pancytokeratin (F) and WT1 (G). A high proliferative index is demonstrated by Ki-67 labelling in approximately 95%-100% of tumour cells (H).

Later, in view of high serum creatinine (5.5 mg/dL) and obstructive symptoms, including facial puffiness and generalised oedema, he underwent bilateral percutaneous nephrostomy insertion under ultrasound guidance (Figure 2). Ultrasound-guided core needle biopsy was taken from the lesion. Histopathology showed features of a small round cell tumour of the urinary bladder (Figure 3). Immunohistochemistry (IHC) examination revealed strong positivity for CD20 and c-MYC, and negative for CD3, TdT, desmin, pancytokeratin and WT1, with proliferative index Ki-67 of approximately 95%-100%, indicative of high-grade B-cell NHL (Figure 4).

Bone marrow trephine biopsy showed no marrow infiltration. Positron emission tomography-computed tomography (PET-CT) revealed no evidence of distant metastasis. He was referred to the paediatric oncology department for chemotherapy. He was classified as stage IIIB and received one cycle of COP (cyclophosphamide, oncovin and prednisone) for tumour reduction, followed by one cycle of COPDAM (cyclophosphamide, oncovin, prednisone, doxorubicin (Adriamycin) and methotrexate). Follow-up contrast-enhanced computed tomography (CECT) of the abdomen demonstrated more than 60% reduction in tumour bulk. The current treatment plan is to continue chemotherapy as per the French-American-British (FAB) protocol.

## Discussion

In 1885, Eve and Chaffey were the first to report a case of primary malignant lymphoma arising from the lymphatic tissue of the urinary bladder [3]. Generally, the urinary bladder is devoid of lymphoid tissue, so primary NHL is a rare site for urinary bladder, and it accounts for less than 1% of all bladder malignancies and represents approximately 0.2% of all extra-nodal lymphoma cases [4]. The most frequently reported symptoms in such patients are gross haematuria, urinary tract infections, dysuria, increased frequency of urination and abdominal or back pain [5]. In our case, the patient presented with increased urinary frequency and urgency. Due to the rarity of primary NHL in the urinary bladder, the aetiology has not been elucidated [6]. As described by Cohen et al., malignant lymphoma of the urinary bladder can be categorised into three types: (a) localised primary bladder lymphoma, (b) disseminated non-localised lymphoma initially presenting in the bladder and (c) secondary lymphoma involving the bladder in patients with a prior history of the disease [7]. In our case, the diagnosis falls under the category of localised primary bladder lymphoma, as recurrent or disseminated disease was excluded based on PET-CT imaging.

The imaging appearance of urothelial tumours and NHL of the urinary bladder on ultrasound and CECT is often similar, making definitive diagnosis reliant on histopathological evaluation combined with IHC [8]. In our case, ultrasound and CECT were utilised for tumour localisation and assessment of local invasion, while the final diagnosis was confirmed through histological analysis and IHC. The most common histological types are small B-cell lymphocytic lymphoma and diffuse large B-cell lymphoma (DLBCL). Cells routinely express CD20, CD79a and bcl-2 and are variably positive for CD10, CD5 and bcl-6 [5]. In our case, tumour cells did not express cytokeratin and bcl-2 but were positive for CD20, CD45 and vimentin, which was more in favour of Burkitt lymphoma. Low-grade lymphomas typically express CD20, CD21 and CD43 cell markers, while high-grade lymphomas commonly test positive for CD20 and CD3 [9]. After type identification, the next important step is to assess the aggressiveness of the disease by proliferative index (Ki-67) for the purpose of initiating the most suitable treatment regimen for that patient. In our case, proliferative index (Ki-67) positivity was 95%, which shows its aggressive nature.

Once lymphoma is identified as a high-grade tumour, it is essential to rule out systemic involvement. This can be achieved through a bone marrow biopsy and PET-CT scan [6]. In our case, systemic disease was excluded using a PET-CT scan. Treatment options for primary NHL of the urinary bladder include surgery, radiotherapy and chemotherapy [2]. In our case, surgery was not necessary; chemotherapy alone led to significant improvement in urinary symptoms and a marked reduction in tumour size. The R-CHOP regimen (rituximab, cyclophosphamide, hydroxydaunorubicin (doxorubicin), oncovin (vincristine) and prednisone) is the most commonly used chemotherapy protocol, with a high reported success rate for both low-grade and high-grade primary bladder lymphomas [5]. Our patient received one cycle of COP followed by one cycle of COPDAM. The plan is to continue chemotherapy according to the French-American-British (FAB) protocol.

## Conclusions

Primary NHL of the urinary bladder is an extremely rare neoplasm in the paediatric age group. Diagnosis is established based on characteristic histopathological features and confirmed through immunohistochemical analysis. Chemotherapy remains the mainstay of treatment and offers favourable outcomes, even in cases of high-grade and locally advanced disease.
